# Frontal Mucocele following Previous Facial Trauma with Hardware Reconstruction

**DOI:** 10.1155/2016/4236421

**Published:** 2016-11-28

**Authors:** Megan EuDaly, Chadd K. Kraus

**Affiliations:** Department of Emergency Medicine, University of Missouri, Columbia, MO 65212, USA

## Abstract

Mucoceles are cysts that can develop after facial bone fractures, especially those involving the frontal sinuses. Despite being rare, mucoceles can result in serious delayed sequelae. We present a case of a frontal mucocele that developed two years after extensive facial trauma following a motor vehicle crash (MVC) and review the emergency department (ED) evaluation and treatment of mucocele. Early recognition, appropriate imaging, and an interdisciplinary approach are essential for managing these rare sequelae of facial trauma.

## 1. Introduction and Case Presentation

A 21-year-old female presented to the emergency department (ED) with an approximately 10-day history of frontal headache and a “boggy” forehead, with associated photophobia and phonophobia. Raising her eyebrows was difficult due to swelling and tenderness above her nose, and she reported nasal congestion and mild rhinorrhea. She also noted subjective fevers. She had noticeable worsening of right eye diplopia over the last few days. Past history was significant for MVC two years earlier with multiple facial fractures that required facial reconstruction of the nasal bones, the frontal sinus with obliteration and a pericranial flap, the right orbital floor, the right zygoma, and nasal septal fixation with hardware.

**Figure 1:**
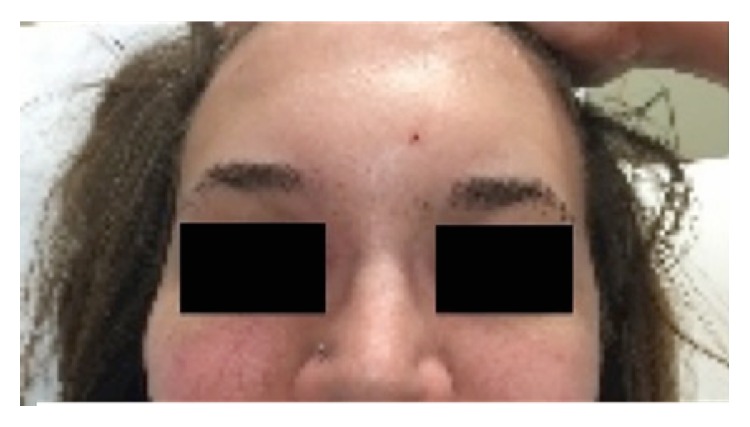
Patient photo showing forehead swelling.

At time of ED presentation, vital signs were normal and the patient was afebrile. On physical exam, there was noticeable swelling and fluctuance over the glabellar region of her forehead (Figure 1: patient photo) with tenderness to palpation over the forehead and nose. The patient's extraocular movements were intact and her vision was grossly normal. She reported diplopia when she looked superiorly and inferiorly with her right eye. No erythema, bruising, drainage, neurologic deficit, ptosis, or exophthalmos was noted.

**Figure 2:**
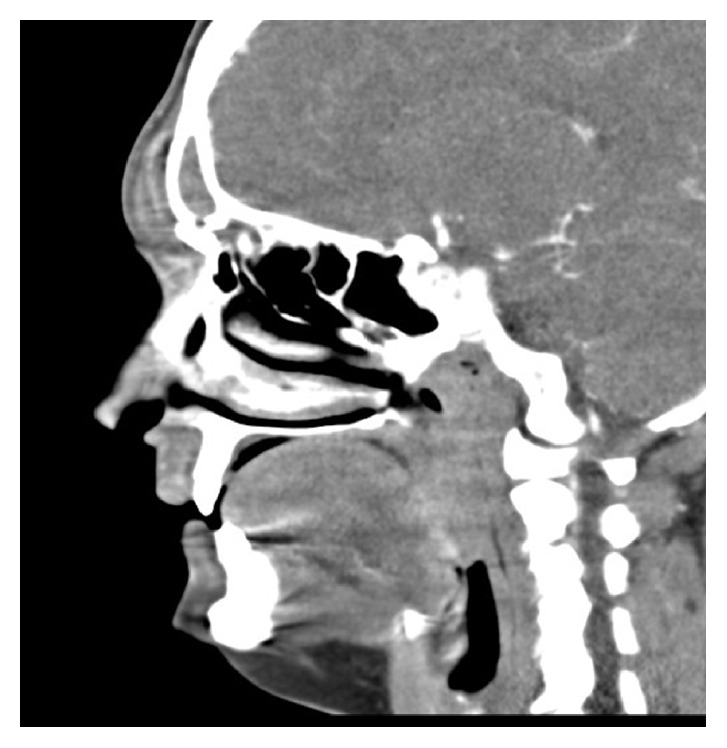
Sagittal CT image (mucocele overlying frontal sinus).

Labs were unremarkable except for leukocytosis of 14,000. Contrast-enhanced computed tomography (CT) scan of the head and maxillofacial structures revealed a 2.3 cm transverse by 4 mm anteroposterior phlegmon versus early abscess along with localized, reactive lymphadenopathy (Figure 2: sagittal CT image). Complete opacification of bilateral frontal sinuses was noted. Given the patient's surgical history, plastic surgery was consulted and needle aspiration performed with culture to evaluate for a mucocele versus a mucopyocele. The patient was admitted to the hospital from the ED for IV antibiotics (vancomycin and piperacillin/tazobactam). The patient was subsequently discharged three days later on sulfamethoxazole/trimethoprim with clinic follow-up scheduled.

Four days after hospital discharge, the patient was readmitted from the clinic with headaches, blurred vision, and worsening forehead swelling. Surgery was performed one week after initial ED presentation. Previous frontal bone hardware was removed with anterior table osteotomy and central galeal flap to the frontal sinus. Postoperative CT showed improvement of soft tissue swelling over anterior forehead. Following the procedure, blurred vision and pain had resolved and future surgical fixation of frontal sinus table was pending follow-up disposition.

## 2. Discussion

Mucoceles develop from accumulation of mucoid secretions in the epithelial lining of the sinuses [1]. Expansion and destruction of the sinuses can occur with slow growth and typically occur as a result of inflammation, surgery, infection, trauma, or history of sinusitis [1, 2]. Up to 28 percent of mucocele cases are related to trauma [2]. Mucoceles that become infected are called mucopyoceles and most commonly develop in the frontal sinuses [1–3].

In patients presenting to the ED with headaches, facial swelling, vision changes, dizziness, proptosis, swelling, rhinorrhea, exophthalmos, or other concerning symptoms and a history of facial bone trauma or surgical repair with or without hardware, there should be a high clinical suspicion for mucocele or mucopyocele [1, 2]. These patients are at risk for delayed sequelae, years or even decades after trauma, including sepsis, encephalitis, meningitis, brain abscess, osteomyelitis, cavernous sinus thrombosis, and meningitis [1–5]. In the ED, these patients should undergo early advanced imaging (e.g., CT or magnetic resonance imaging) [1] and the emergency physician should consult with a multidisciplinary surgical team such as otolaryngology or plastic surgery [1, 4, 5]. Additionally, evaluation and treatment for possible sepsis, including early, broad-spectrum antibiotic administration, should be considered.
